# Subclinical Enteric Parasitic Infections and Growth Faltering in Infants in São Tomé, Africa: A Birth Cohort Study

**DOI:** 10.3390/ijerph15040688

**Published:** 2018-04-05

**Authors:** Marisol Garzón, Luís Pereira-da-Silva, Jorge Seixas, Ana Luísa Papoila, Marta Alves

**Affiliations:** 1 Tropical Clinic Teaching and Research Unit, Instituto de Higiene e Medicina Tropical, Universidade NOVA de Lisboa; 1349-008 Lisbon, Portugal; garzon.marisol1@gmail.com (M.G.); JSeixas@ihmt.unl.pt (J.S.); 2 Global Health and Tropical Medicine R&D Center, Instituto de Higiene e Medicina Tropical, Universidade NOVA de Lisboa; 1349-008 Lisbon, Portugal; 3 Medicine of Woman, Childhood and Adolescence Teaching and Research Area, NOVA Medical School, Universidade NOVA de Lisboa; 1169-056 Lisbon, Portugal; 4 Research Unit, Centro Hospitalar de Lisboa Central; 1169-045 Lisbon, Portugal; ana.papoila@nms.unl.pt (A.L.P.); marta.alves@chlc.min-saude.pt (M.A.)

**Keywords:** birth cohort, enteric parasitic infection, infant growth, low-middle-income country, subclinical infection

## Abstract

The associations between enteric pathogenic parasites and growth in infants in São Tomé were explored using a refined anthropometric approach to recognize early growth faltering. A birth cohort study was conducted with follow-up to 24 months of age. Microscopic examination for protozoa and soil-transmitted helminths was performed. Anthropometric assessments included: z-scores for weight-for-length (WLZ), length-for-age (LAZ), weight (WAVZ) and length velocities (LAVZ), length-for-age difference (LAD), and wasting and stunting risk (≤−1 SD). Generalized additive mixed effects regression models were used to explore the associations between anthropometric parameters and enteric parasitic infections and cofactors. A total of 475 infants were enrolled, and 282 completed the study. The great majority of infants were asymptomatic. *Giardia lamblia* was detected in 35.1% of infants in at least one stool sample, helminths in 30.4%, and *Cryptosporidium* spp. in 14.7%. *Giardia lamblia* and helminth infections were significantly associated with mean decreases of 0.10 in LAZ and 0.32 in LAD, and of 0.16 in LAZ and 0.48 in LAD, respectively. *Cryptosporidium* spp. infection was significantly associated with a mean decrease of 0.43 in WAVZ and 0.55 in LAVZ. The underestimated association between subclinical parasitic enteric infections and mild growth faltering in infants should be addressed in public health policies.

## 1. Introduction

*Giardia lamblia, Cryptosporidium* spp. and soil transmitted helminth (STH) infections affect the world’s poorest population. Among these parasites, only STH are included in the current World Health Organization (WHO) list of neglected tropical diseases (NTD), although *Giardia* and *Cryptosporidium* also share the characteristics of NTD [1]. The comprehensive multinational Malnutrition and Enteric Disease (MAL-ED) study [2], carried out in eight low and middle-income countries (LMIC), highlighted the role of protozoans as etiologic agents of enteric infections in the first two years of age. Several other birth cohort studies in LMIC have provided evidence that *Giardia lamblia* [3,4], *Cryptosporidium* spp. [5,6], and STH [7,8] infections during the first two years of age have an impact on growth, whether or not in association with diarrhea [9]. In these settings, the importance of exposure to enteric pathogens has been hypothesized not solely based on the existence of diarrhea, raising the question of their role in causing “hidden” damage, which could result in growth shortfalls [9,10].

Refined anthropometric monitoring of infants at risk of growth faltering can provide information on otherwise unobserved changes in disease exposure [11]. A comprehensive approach to growth monitoring should include not only the conventional z*-scores* for attained growth [12], but also the growth velocity [13]. While the attained growth at a single point in time is a cumulative measure of growth rate, velocity measures display greater sensitivity in capturing influencing factors and have greater potential in predicting short-term changes in growth [14]. The height-for-age difference has also been proposed as a complementary and more adequate measurement to assess changes in height over time [15]. Furthermore, the use of a lower cut-off of ≤−1 standard deviation (SD) is advisable to detect infants at risk of undernutrition [16]. 

This study aimed to explore the association between enteric pathogenic parasites and growth in infants from a birth cohort in São Tomé. We hypothesize that neglected subclinical enteric parasitic infections during the first 24 months of age are associated with early growth faltering.

## 2. Materials and Methods

This study is nested within a birth cohort study aimed at determining the association between enteric parasitic infections and several outcomes in infants living in São Tomé, the principal island of the Republic of São Tomé and Príncipe (STP), a LMIC situated in the Gulf of Guinea, off the western equatorial coast of Central Africa. Details of the birth cohort are described elsewhere [17]. The study protocol was approved by the ethical committee of the Ministry of Health; informed consent was obtained from the legal tutors of all infants, and the study was conducted in accordance with the principles of the Declaration of Helsinki. Briefly, 500 appropriate-for-gestational age infants within the first 28 postnatal days were eligible. Neonates were recruited at the main health care centers at the outpatient facility “Protecção Materno-Infantil” (Maternal and Child Protection) in Agua Grande district, and at the local hospitals in Lembá and Caué districts. Those with low birth weight (less than 2500 g), born preterm (less than 37 weeks of gestation), without gestational age information, and with major congenital malformations were excluded. Infants were enrolled from March to June 2013 and followed-up to 24 months of age. The study was considered complete for infants who were assessed at 24 months of age and attended at least 10 (50%) of all scheduled points of assessment. 

Data collection included epidemiological, socioeconomic, and feeding practices variables and clinical data. In the first scheduled visit, a questionnaire on demographic and socioeconomic status was applied to mothers. Further on, at each point of assessment, feeding practices and clinical events were enquired, anthropometric measurements recorded, and stool samples collected for parasite examination. All these procedures were performed by the same trained observer (MG).

### 2.1. Socioeconomic Status

The multidimensional poverty index (MPI) that includes education, health, and standard of living dimensions [18] was used to assess the socioeconomic household status. In the original MPI definition, the education dimension includes years of schooling and child enrolment; health dimension includes child mortality and nutrition; and the standard of living dimension includes electricity, drinking water, sanitation, flooring, cooking fuel, and assets [18]. Each dimension is equally weighted, and each indicator within a dimension is also equally weighted. A household is identified as “multidimensionally poor” if MPI score is ≥33.3% and ‘’severely deprived’’ if MPI score is ≥50% [18]. As in a previous large-scale study [19], we have adapted the MPI evaluation based on variables directly affecting infant growth. In the education dimension, we only included the mother education, considering that having no formal education or only basic level (primary school) education is a risk factor for stunting [20]; a deprived household was defined if the mother had less than five years of school education. In the health dimension, even if the original MPI includes nutrition, we only considered child mortality, since the nutritional status is our main outcome variable [19]. The standard of living dimension was incorporated according to the original MPI definition [18].

### 2.2. Feeding Practices 

The questionnaire on feeding practices used the United Nations Children’s Fund (UNICEF) indicators [21], including the variables: exclusive breastfeeding at 6 months, continued breastfeeding at 1 year of age, continued breastfeeding at 2 years of age, infants ever breastfed, and age of introduction of solid, semi-solid, or soft foods. 

### 2.3. Clinical Data

Clinical data included acute infectious events observed at the time of scheduled visits, as well as events not observed but reported to have occurred within the two preceding weeks. Clinical events included acute diarrhea (either watery or bloody diarrhea, lasting less than 14 days), persistent diarrhea (lasting more than 14 days) [22], acute respiratory infections including pneumonia, other respiratory infections [23], and malaria confirmed by Rapid Diagnostic Test and/or thick blood smear microscopic identification. Other noninfectious and chronic conditions were also recorded.

### 2.4. Parasite Examination Techniques

Stool samples were collected for intestinal parasites examination in seven points of assessment, at 3, 6, 9, 12, 16, 18, and 24 months of age. The examination techniques are described in detail elsewhere [17]. Briefly, microscopic ova and parasite examination was performed in iodine-stained wet mounts of feces dissolved in saline and after formol-ether concentration procedure [24]. A cold acid-fast Kinyoun stain (Biomerieux^®^, Marcy-l’Étoile, France) was used for *Cryptosporidium* spp. and coccidian species (*Cystoisospora* and *Cyclospora*) detection. A Rapid test for *Giardia duodenalis* detection (STICK Giardia/simple Giardia Operon, Immune and Molecular diagnostics) was used for liquid stool samples. Each microscopic examination was double checked by two independent trained observers. It should be noted that after delivery of stool samples at each point evaluation, infants older than 1 year received mebendazole every four months, in compliance with the WHO preventive chemotherapy strategy for STH [25] implemented in São Tomé and Principe. Additionally, infants were treated for *Giardia lamblia* with metronidazole in case of microscopic detection of trophozoites (regardless of symptoms), detection of cysts, or a positive rapid test in symptomatic infants, according to the current recommendations [26].

### 2.5. Anthropometry

Anthropometry was performed monthly during the first year of age and bimonthly during the second year. Baseline neonatal measurements between the 1st and 28th postnatal day were recorded. Anthropometric measurements were done according to WHO-recommended techniques [12]. Infants were weighed using an electronic infant scale (Seca 334, GmbH & Co. KG, Hamburg, Germany) to the nearest decigram, and crown-heel length was measured using an infantometer (Seca 207, GmbH & Co. KG, Hamburg, Germany) to the nearest millimeter. Attained growth included body weight (kg) and length (cm) measurements converted into the respective age- and sex specific *z-*scores (WLZ) and (LAZ) using the WHO Anthro software v.3.2.2 (WHO, Geneva, Switzerland). Absolute length-for-age difference (LAD) was calculated by subtracting the measured length to the reference age- and sex-specific median value and expressed in absolute value (cm) [15]. Growth velocity was based on two-month interval weight and length increments, which were converted into respective age and sex specific *z-*scores for weight (WAVZ) and length (LAVZ), following the WHO methodology [13]. Risk of wasting and stunting were defined as WLZ and LAZ ≤ −1 SD > −2, and moderate-to-severe wasting and stunting as WLZ and LAZ ≤ −2 SD, respectively.

Mother’s height was measured following standard procedures using a stadiometer (Seca 213, GmbH & Co. KG, Hamburg, Germany) to the nearest 0.1 cm.

## 3. Statistical Analysis

The MPI score, feeding practices, clinical data, anthropometric measures, and parasitological results are described with frequencies (percentages) and with mean (SD) or median (min-max), as appropriate. In the univariable regression analysis, all the variables with a *p*-value < 0.25 were selected for the multivariable models. Generalized additive mixed effects regression models were used to take into account the correlation structure between measures in time, and to explore the association of each anthropometric parameter with enteric parasitic infections and other relevant cofactors (MPI score, maternal stature, feeding practices, and acute clinical events). For all multivariable models, age was modelled with splines, because a non-linear association with each anthropometric parameter was identified. A level of significance α = 0.05 was considered. Data were analysed using Stata (Stata Statistical Software: Release 13. StataCorp LP: College Station, TX, USA).

## 4. Results

From 500 eligible neonates, 25 were excluded due to preterm birth, low birth weight, major congenital malformation, and perinatal asphyxia. Thus, 475 infants were enrolled in the birth cohort, corresponding to approximately 8.6% of live-births in São Tomé in 2012 [27]. 

**Figure 1:**
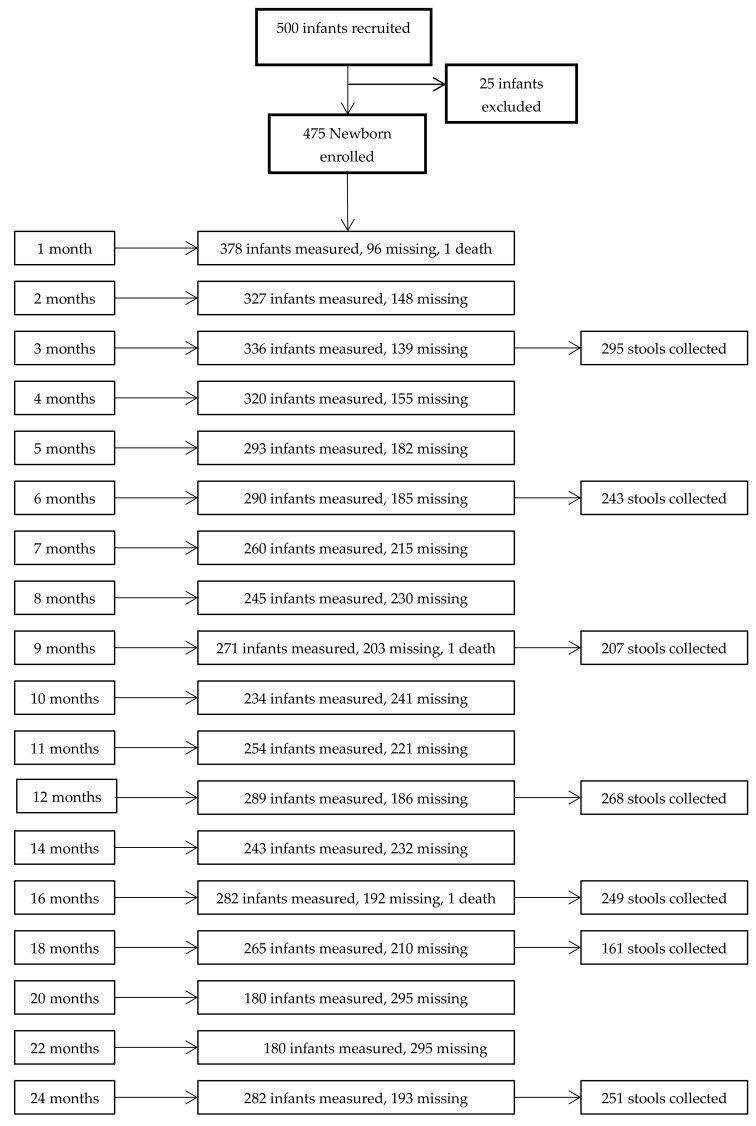
Flow-chart with the number of evaluated patients and number of stool samples collected at each point of assessment.

During the study period, different proportions of infants missed the scheduled visits, corresponding to an attrition rate of 41.05% at 24 months (Figure 1). Nevertheless, many of the infants that were not present at one point of assessment returned for the next. Two hundred eighty-two (59.4%) infants completed the study, attending a median of 18 points of assessment. Significant differences were found between infants who completed 24 months of follow-up and infants who prematurely dropped-out (Supplementary Table S1). Infants completing the study had higher weight and length at birth, belonged predominantly to Agua Grande district (higher wealth index), had better access to improved sanitation and water source, and their mothers were older and received longer education.

The Figure 1 includes a flow-chart with the number of infants with anthropometric measurements and the number of stool samples collected at the different points of assessment.

Results for socio-demographic variables, socioeconomic status, and feeding practices are presented in Table 1.

### 4.1. Socioeconomic Status 

From the 475 households enquired, only 287 (60.8%) provided data for MPI scoring. In the education dimension, 53.7% of mothers had less than 5 years of school; in the health dimension, child mortality occurred in 2.8% of households; and in the living standard dimension, 13.9% of households were deprived of electricity, 33.8% of improved sanitation, 0.3% of improved water source, 0.7% of finished floor, 10.1% of cooking with solid fuel, and 24.4% of assets. Based on the total MPI score, 24.0% of households were classified as deprived, and of these, one third was severely deprived.

### 4.2. Feeding Practices 

The great majority (88.4%) of infants were exclusively breastfed during the first 6 months of age. After this age, 64.8% and 13.9% of infants maintained breastfeeding up to 1 year and 2 years of age, respectively. Complementary feeding was introduced at a mean age of 6 months.

### 4.3. Clinical Data

The frequency of acute diarrhea episodes was less than 5.2% during the first 5 months of age, and subsequently it increased, varying from 6.8% to 15.1%; most episodes consisted in watery diarrhea and very few were bloody, persistent, or complicated by dehydration. The frequency of acute respiratory infections was less than 3.2% during the first 2 months of age, and subsequently it increased, varying from 19.9% to 32.8%; most were upper respiratory infections. Malaria was diagnosed in four infants, only after 12 months of age. HIV test was positive in one infant. Other chronic conditions recorded were allergic symptoms in 174 infants and anemia in 133 (6 with sickle cell anemia). During the study period, three deaths occurred with clinical diagnoses (without autopsy) of heart failure at age of 1-month, acute hemorrhagic syndrome due to poisoning at age of 13 months, and pneumonia at age of 16 months.

### 4.4. Enteric Parasites

During the study period, 1674 stool samples were collected from 388 (81.7%) infants. From these, stool samples were obtained from 220 (56.7%) infants in at least four of the seven scheduled collections. The frequencies of intestinal pathogenic parasites found at each point of assessment are shown in Table 2. Enteric parasites were not detected at 3 months of age; subsequently, the frequency of parasitic infection increased progressively with age, from 4.9% at 6 months to 43.8% at 24 months. Single parasitic infections predominated. Co-infections were detected from 12 months of age on, and their frequency increased up to 24 months of age. The three most frequent pathogenic parasites found, either as single or multiple infections, were by decreasing order *Giardia lamblia*, STH, and *Cryptosporidium* spp. (Table 2). *Entamoeba histolytica*/*dispar* complex was not observed. In 41.2% of infants, no enteric parasite was detected at any point of assessment. 

*Giardia lamblia* was detected in 35.1% of infants in at least one stool sample. Detection rate increased with age, with an incidence ranging between 2.5% and 5.3% from 3 to 11 months of age, and between 14.9% and 23.9% from 12 to 24 months of age (Table 2). The median age of first detection was 16 months. Twenty-two percent of infants had one episode of *Giardia* infection, 10.9% two episodes, and 1.8% three or more episodes, either as single or multiple-agent infection (Table 3). 

*Cryptosporidium* spp. was detected in 14.7% of infants in at least one stool sample. Its prevalence ranged between 2.5% and 4.8% from 3 to 11 months of age and between 3.2% and 7.1% from 12 to 24 months of age (Table 2). The median age of first detection was 12 months. Thirteen percent of infants had one episode of *Cryptosporidium* spp. infection, and 1.1% had two or more episodes, either as single or multiple-agent infection (Table 3). 

STH were observed in 30.4% of infants in at least one stool sample. Detection rate increased with age, with no cases detected up to 6 months of age, and from 12 to 24 months it ranged between 12.3% and 27.5% (Table 2). The median age of first detection was 16 months. Eighteen percent of infants had one episode of STH infection, 8.3% two episodes, and 3.8% three or more episodes, either as single or multiple-agent infection (Table 3). 

### 4.5. Anthropometry

Attained growth data were based on 5338 longitudinal observations during the study period (Supplementary Table S2). 

A mean weight of 3.34 (0.46) kg in females and 3.50 (0.49) kg in males, and a mean length of 49.7 (1.89) cm in females and 50.7 (1.86) cm in males, were obtained in the neonatal period. Expressed as *z-*scores, these values corresponded to 0.03 and −0.04 for WLZ, and −0.73 and −0.68 for LAZ for females and males, respectively. After the neonatal period, the WLZ and LAZ were > −1 SD, with slight fluctuations up to 24 months of age. At 24 months of age, females had a mean weight of 11.08 (1.12) kg and males of 11.64 (1.13) kg, with a mean length of 84.7 (3.25) cm and 86.4 (3.1) cm, respectively. Expressed as *z*-scores, these values corresponded to 0.14 and −0.23 for WLZ, and −0.55 and −0.49 for LAZ for females and males, respectively. 

The mean LAD in the neonatal period was −1.52 cm in females and −1.44 cm in males. This length difference remained almost unchanged during the study period, with −1.52 cm in females and −1.33 cm in males at 24 months of age (Supplementary Table S2).

Growth velocity data were based on 2514 two-month interval observations. During the study period, the mean WAVZ ranged from −0.66 to 0.33. The mean LAVZ was −1.34 in the first interval (from 0 to 2 months of age), and subsequently increased ranging from −0.41 to 0.27 (Supplementary Table S2).

In the neonatal period, 13.3% were at risk of wasting (WLZ ≤−1 SD >−2 SD), and 3.3% moderately-to-severely (WLZ ≤−2 SD) wasted; 29.7% were at risk of stunting (LAZ ≤−1 SD >−2SD), and 7.8% moderately-to-severely (LAZ ≤−2 SD) stunted. At 24 months of age, 14.4% of infants were at risk of wasting, and 0.7% moderately-to-severely wasted; 19.9% were at risk of stunting, and 8.9% moderately-to-severely stunted. 

### 4.6. Enteric Parasite Infections and Growth 

The univariable analysis for attained growth (WLZ, LAZ, and LAD) and growth velocity (WAVZ and LAVZ) are presented in Supplementary Tables S3 and S4, respectively.

The adjusted associations between variables found in multivariable analysis are presented in Table 4. In relation to attained growth, no association was observed between WLZ and any parasitic infection; regarding covariables, the WLZ was positively associated with exclusive breastfeeding (*p* < 0.001) and negatively with the MPI score (*p* = 0.005) and acute diarrhea (*p* < 0.001). The LAZ was negatively associated with *Giardia lamblia* (*p* = 0.018) and STH (*p* < 0.001) infections; regarding covariables, the LAZ was positively associated with exclusive breastfeeding (*p* = 0.003) and mother’s height (*p* < 0.001), and negatively with the MPI score (*p* < 0.001). LAD was negatively associated with *Giardia lamblia* (*p* = 0.013) and STH (*p* < 0.001) infections; regarding covariables, the LAD was positively associated with exclusive breastfeeding (*p* = 0.006) and mother’s height (*p* < 0.001), and negatively with the MPI score (*p* < 0.001). In relation to growth velocity, WAVZ was negatively associated with *Cryptosporidium* spp. (*p* = 0.023), and with acute diarrhea (*p* < 0.001). The LAVZ was negatively associated with *Cryptosporidium* spp. (*p* = 0.005), and positively with mother’s height (*p* = 0.002).

## 5. Discussion

This birth cohort study conducted in Sao Tomé confirmed the previously described association between enteric pathogenic parasites and growth faltering in infants living in a LMIC [3,4,5,6,7,8].

### 5.1. Enteric Parasites

Our study is the first longitudinal study on the prevalence of enteric parasites in infants in São Tomé. A high prevalence was found, with at least one parasite detected in 58.5% of infants in the first 24 months of age. The three most frequently detected pathogenic parasites, either as single or multiple infections, were *Giardia lamblia*, STH, and *Cryptosporidium* spp. The frequency of detection of *Giardia lamblia* and *Cryptosporidium* spp. in our study was similar to that reported in infants from LMIC in the multinational study MAL-ED [2], but the prevalence of STH in our cohort was much higher. Previous studies in STP have reported a high overall prevalence of enteric parasites, affecting 86.7% of preschool children [28,29] and 64.7% of primary school children [30,31]. These studies found a 56.3% prevalence of *Ascaris lumbricoides*, 52.5% of *Trichuris trichiura*, 41.7% of *Giardia lamblia* [29], and 8.9% of *Cryptosporidium* spp. [28]. Our study confirmed that this country is highly endemic for enteric parasites, infecting or colonizing infants from early ages, mainly with *Giardia lamblia* and STH. The main factor associated with this high prevalence is probably related to limited access to improved sanitation in the households [32]. 

In this study, enteric parasites were detected mostly in infants without diarrhea. In LMIC, there is growing evidence that *Giardia lamblia* [3,4] and *Cryptosporidium* spp. [6,33] are etiologic agents of enteric infections in infants without overt diarrhea [2,34]. Several factors may explain the excretion of enteric pathogens in the absence of diarrhea, related either to the pathogen (strain virulence, prolonged excretion of cysts), to the host (immune response, nutritional status, microbiota), and/or to the environment [35]. In highly endemic settings, repeated infections by *Giardia* and STH seem to result in an asymptomatic clinical status, possibly reflecting acquisition of an immunoregulatory host environment [36,37]. Hence, one may speculate that in infants living in São Tomé, the continuous exposure to enteric parasites may have induced an immune response capable of preventing clinical illness but not intestinal colonization [35,36,37]. In addition, the high proportion of exclusively breastfeed infants in our cohort may have played a role in protecting against more severe forms of diarrhea or even diarrhea at all [35,38].

### 5.2. Infant Growth

The refined anthropometric approach we used for growth monitoring allowed the identification of subtle deficits that would have been missed if relying solely on conventional metrics and thresholds [16]. We employed metrics complementing the conventional z-scores for growth, including (i) growth velocity, since it accurately reflects the effect of current events [14]; (ii) LAD, as an alternative metric to assess changes in height over time [15]; and (iii) the cut-off ≤ −1 SD to define wasting and stunting in order to include mild (or at risk of) and moderate-to-severe undernutrition [16]. In our infants, the values of WLZ and LAZ from birth to 24 months of age were close to WHO standards [12]. However, when length was expressed as LAD [15], the negative differences became evident from the neonatal period (around −1.5 cm), and these negative values remained practically unchanged up to 24 months of age. The length velocity (LAVZ) in the first interval showed negative values (−1.34), although with subsequent catch-up to values close to WHO standards [13]. The weight velocity z-scores (WAVZ) were within normal range during the 24 months of age. Our results reflect that linear growth (LAZ, LAD, LAVZ) was more affected than weight (WLZ, WAVZ). It is noteworthy that a deficit in length was observed at the neonatal period, probably reflecting intrauterine growth restriction; no further deterioration was noticed after birth. Finally, we found a much lower overall prevalence of moderate-to-severe stunting (7.8%) in comparison to the 17.2% value described in STP for children under-5 [39]. As reported [40], the use of the cut-off ≤−1 SD allowed detection of an important proportion of infants at risk of undernutrition (mild degree), which was far higher than those with moderate-to-severe degrees. The recognition of infants at risk of undernutrition is important from a public health perspective, since it provides information on otherwise unobserved changes in disease exposure [11,41].

### 5.3. Enteric Parasites and Growth Faltering

The quantification of the individual impact of subclinical infections by enteric parasites on growth has been less explored than in infected children with diarrhea [10]. In our study, the multivariable analysis showed significant associations between *Giardia lamblia* and STH infections and deficit in linear attained growth (LAZ and LAD) and between *Cryptosporidium* spp. infection and deficit in growth velocity (WAVZ and LAVZ). Several clinical and epidemiological studies corroborate our findings. The association between *Giardia lamblia* infection and growth restriction is well documented in infants from LMIC [3,4,42]. In the MAL-ED study, a strong association between persistent *Giardia* infection in the first six months of age and length deficit at 2 years was found [4]. In Bangladesh, the presence of *Giardia* in the first 6 months of age was associated with a decrease in LAZ but not WAZ at 2 years of age [3]. Studies addressing the impact of STH infections on infant growth are much scarce [7,8,43]. In Peru, reduced LAZ was observed in infants with moderate to heavy helminth infections compared with those not infected [7]. In a Kenyan birth cohort, *Ascaris* infection at 24 months of age was significantly associated with a decrease in LAZ [8]. The association between *Cryptosporidium* spp. and growth restriction in infants was also reported in longitudinal studies [5,6,44,45]. For instance, Bangladeshi infants with *Cryptosporidium* spp. infection had a 2-fold increased risk of stunting at 2 years of age [6]. In our study, *Giardia* and STH infections affected linear growth but not weight, which can reflect a cumulative, long-term sustained exposure to these pathogens in this endemic country [46]. In the particular case of STH, the association we found with growth deficit was mostly due to *Ascaris lumbricoides*, since *Trichuris trichiura* was detected in less than 3% of infants, and no hookworms were detected. Noteworthy, the deleterious associations between these parasites and growth were observed even though infants received regular deworming therapy [25], probably indicating a very high frequency of re-infection. The specific association of *Cryptosporidium* spp. infection with low growth velocity rate, but not with attained growth, may reflect acute transient rather than persistent infection in the relatively immune, competent infants of our cohort, who were HIV-negative, less severely undernourished, and benefited from the protective effect of specific antibodies in breast milk [47].

The physiopathology of growth faltering in enteric parasitic infection could be related to several mechanisms, including microbial-driven nutrient deficiencies, intestinal inflammation, gut dysfunction, and increased intestinal permeability [48]. In experimental models, *Giardia lamblia* and *Cryptosporidium* spp. infections cause epithelial disarrangements, blunted villus architecture, and chronic inflammation, resulting in malabsorption and poor weight gain [49,50]. Jejunal biopsies of *Ascaris*-infected children also showed histological changes in villi, crypts, and lamina propria, affecting the intestinal absorptive capacity [51]. Clinical studies have reported increased gut permeability in *Giardia* [9,52], *Cryptosporidium* spp. [53], and helminth infections [54]. In a subsample of our cohort, we found significant association between increased intestinal permeability and undernutrition, although not between enteric parasitic infections and gut inflammation or increased permeability [17]. Together, experimental and clinical evidence suggest that enteric protozoa and STH may be considered as “stunting” pathogens when associated with diarrhea [55], and in our opinion this concept should be extended to subclinical infections. Affected infants may have a limited capacity to repair mucosal damage, which in turn could contribute to malabsorption, disturbed nutrient uptake and transport, and increased metabolic needs [56]. These factors, together with the yet poorly understood intestinal host-pathogen-microbiome interactions, apparently result in a negative impact on growth, thus jeopardizing the achievement of their full potential [9,56]. The combination of subclinical parasitic infections with subtle growth faltering detected in our infants is of utmost importance, since it can easily be unrecognized unless the adequate screening tools are used, resulting in a missed opportunity of targeting this at-risk population in public health policies.

In our study, specific cofactors were associated with better growth. Exclusive breastfeeding was associated with enhanced attained growth. This may be in relation to the high proportion (88.4%) of infants exclusively breastfed at 6 months of age, when compared with the 37% value reported in infants from LMIC [57]. Maternal stature was another protective factor for attained growth and growth velocity. In LMIC, it is reported that each 1-cm increase in maternal height predicted an increase in 0.037 in LAZ at two years of age [58]. Conversely, specific cofactors were associated with inadequate growth. Poor wealth status reflected by a higher MPI score was associated with a decrease in attained growth, as described in children from LMIC [59]. Although the frequency of acute diarrhea in our cohort was low, it was nevertheless significantly associated with weight deficit. It was reported that acute episodes of diarrhea are associated with deficit in weight but not in length [60].

The main limitation of this study was the high attrition rate of 40.25%. Infants who completed the study had better anthropometry indicators at birth and better socioeconomic status than those lost to follow-up. This may have biased our results in a more favorable way. The attrition rate effect did not depend on the research team; that is, it was random and lost to follow-up. No important bias is described with levels of loss up to 60% when the missing is related to random mechanism [61]. Furthermore, in our study the loss to follow-up was a sort of wave attrition, i.e., many infants not present at one follow-up visit returned for the next, allowing recapture for analysis [62]. Other limitations are related to the accuracy of reported clinical events that may have been affected by a recall bias and the accuracy of enteric parasites diagnosis relying on microscopic exam. It is accepted that microscopic examination of a single stool sample has low sensitivity [63]. Nevertheless, some factors may have improved the detection rate in our study, including the fact that stool samples were obtained in a highly endemic setting [64] and the use of a concentration technique [24]. 

## 6. Conclusions

This is the first birth cohort study ever performed in São Tomé. Since the vast majority of recruited infants were asymptomatic, we were able to detect the deleterious effect of subclinical enteric parasitic infections on infant growth using a refined anthropometric approach. Our findings are consistent with those described in the literature for LMIC. 

Although the magnitude of that effect on growth was mild, in a highly endemic setting for *Giardia lamblia* and helminthic infections, it may represent an obstacle to the potential full achievement of a considerable number of infants in São Tomé. This should therefore be adequately acknowledged and incorporated in public health policies, so that not only the problem of enteric neglected parasitic diseases but also that of neglected patients is addressed.

## Figures and Tables

**Table 1:** Socio-demographic and household characteristics of the cohort (N = 475).

Variable	N	%
Sex		
Females	244	51.4
Males	231	48.6
District		
Agua Grande	363	76.4
Lembá	75	15.8
Caué	37	7.8
Inhabitants per house (mean)	4.7	
Access to improved water source		
Not improved (river)	9	1.9
Improved	461	97.6
Access to improved sanitation		
Not improved	193	40.9
Improved	277	58.7
Multidimensional poverty index (MPI) (N = 287)		
Deprived household	69	24.0
Non-deprived household	218	75.9
Maternal data		
Maternal education level		
None /primary school	152	32.2
Secondary /higher school	319	67.2
Missing information	1	
Mother’s height		
≤ 1.45 m	3	0.6
1.45–1.59 m	250	52.6
≥ 1.60 m	195	41.0
Not measured	27	5.7
Feeding practices		
Exclusive breastfeeding during the first 6 months of age	420	88.4
Breastfeeding plus formula feeding	53	11.2
Formula feeding	1	0.2
Continued breastfed up to 1 year		
Yes	308	64.8
PlNo	7	1.5
Missing information	160	3.4
Continued breastfed up to 2 year		
Yes	66	13.9
No	249	52.4
Missing information	160	33.7
Age (months) of complementary feeding introduction, mean (SD)	5.9 (0.9)	

**Table 2:** Frequency of intestinal pathogenic parasites by age.

Intestinal Parasites	3 Months N = 295	6 Months N = 243	9 Months N = 207	12 Months N = 268	16 Months N = 249	18 Months N = 161	24 Months N = 251
**Infected, % (n)**	0 (0)	4.9 (12)	14.5 (30)	30.2 (81)	33.3 (83)	41.6 (67)	43.8 (110)
**Single infections, % (n)**	0 (0)	4.9 (12)	14.5 (30)	25.7 (69)	30.1 (75)	32.3 (52)	33.9 (85)
*Giardialamblia*	0 (0)	2.5 (6)	5.3 (11)	12.7 (34)	14.45 (36)	15.52 (25)	17.1 (43)
*Cryptosporidium* spp.	NA	2.5 (6)	4.8 (9)	3.7 (10)	3.2 (8)	3.7 (6)	1.6 (4)
*Entamoeba histolytica/dispar complex*	0 (0)	0 (0)	0 (0)	0 (0)	0 (0)	0 (0)	0 (0)
*Cystoisospora belli*	0 (0)	0 (0)	0 (1)	0.7 (2)	0 (0)	1.2 (2)	0 (0)
*Ascaris lumbricoides*	0 (0)	0 (0)	4.3(9)	7.1 (19)	12.4 (31)	8.1 (13)	12.7 (32)
*Trichuris trichiura*	0 (0)	0 (0)	0 (0)	1.5 (4)	0 (0)	3.1 (5)	1.9 (5)
*Necator americanus/Ancylostoma duodenale*	0 (0)	0 (0)	0 (0)	0 (0)	0 (0)	0 (0)	0 (0)
*Strongyloides stercoralis*	0 (0)	0 (0)	0 (0)	0 (0)	0 (0)	0.6 (1)	0 (0)
*Hymenolepis nana*	0 (0)	0 (0)	0 (0)	0 (0)	0 (0)	0 (0)	0.4 (1)
**Multiple infections, % (n)**	0 (0)	0 (0)	0 (0)	4.5 (12)	3.2 (8)	9.3 (15)	9.95 (25)
*Giardia lamblia* + STH	0 (0)	0 (0)	0 (0)	1.5 (4)	1.2 (3)	3.7 (6)	5.6 (14)
*Cryptosporidium spp*. + STH	0 (0)	0 (0)	0 (0)	2.2 (6)	0.4 (1)	0.6 (1)	1.2 (3)
*Giardia lamblia + Cryptosporidium spp.*	0 (0)	0 (0)	0 (0)	0.75 (2)	0.8 (2)	1.9 (3)	0.4 (1)
*Ascaris lumbricoides + Trichuris trichiura*	0 (0)	0 (0)	0 (0)	0 (0)	0.4 (1)	1.2 (2)	1.9 (5)
*Giardia lamblia* + *Cryptosporidium* spp. *+* STH	0 (0)	0 (0)	0 (0)	0 (0)	0.4 (1)	0 (0)	0 (0)
*Ascaris lumbricoides + Trichuris trichiura +* one protozoa	0 (0)	0 (0)	0 (0)	0 (0)	0 (0)	1.9 (3)	0.8 (2)
Total *Giardia lamblia* (single or multiple), % (n)	0 (0)	2.5 (6)	5.3 (11)	14.9 (40)	16.9 (42)	22.9 (37)	23.9 (60)
Total *Cryptosporidium* spp. (single or multiple), % (n)	0 (0)	2.5 (6)	4.8 (9)	7.1 (18)	5.0 (11)	6.45 (10)	3.2 (8)
Total STH (single or multiple), % (n)	0 (0)	0 (0)	4.35 (9)	12.3 (33)	15.3 (38)	22.4 (36)	27.5 (69)

NA: not available, STH: Soil transmitted helminthes

**Table 3:** Cumulative data on enteric parasitic infections, including age at first detection, total number of infections, and number of episodes of infection.

Enteric Parasitic Infections	Age at First Detection	Total Number of Infections	Number of Episodes of Infection % (n)				
	Median (min–max)	% (n)	1	2	3	4	≥5
Never infected, % (n)		41.2 (160/388)					
Ever infected, % (n)		58.8 (228/388)					
*Giardia lamblia* infections (single or multiple)	16.0 (5.9–24.7)	35.1 (136)	22.4 (87)	10.9 (42)	1.0 (4)	0.5 (2)	0.3 (1)
*Cryptosporidium* spp. infections (single or multiple)	12.2 (5.9–24.1)	14.7 (57)	13.7 (53)	0.8 (3)	0.3 (1)	0.0 (0)	0.0 (0)
Soil-transmitted helminths infections (single or multiple)	16.2 (8.8–24.7)	30.4 (118)	18.3 (71)	8.3 (32)	3.3 (13)	0.5 (2)	0.0 (0)

**Table 4:** Multivariable analysis for attained growth (WLZ, LAZ, and LAD) and growth velocity (WAVZ and LAVZ). Only variables that were significant in the univariable analysis were included into the multivariable analysis.

Anthropometric Variables	β-Estimate (95% CI)	*p* Value
**WLZ**		
Exclusive breastfeeding	0.48 (0.23; 0.73)	<0.001
MPI score	−0.07 (−0.12; −0.02)	0.005
Acute diarrhea	−0.19 (−0.27; −0.12)	<0.001
**LAZ**		
*Giardia lamblia*	−0.10 (−0.18; −0.02)	0.018
STH	−0.16 (−0.25; −0.07)	<0.001
Exclusive breastfeeding	0.39 (0.14; 0.65)	0.003
Mother’s height	0.05 (0.04; 0.07)	<0.001
MPI score	−0.11 (−0.16; −0.06)	<0.001
**LAD**		
*Giardia lamblia*	−0.32 (−0.57; −0.07)	0.013
STH	−0.48 (−0.76; −0.20)	<0.001
Exclusive breastfeeding	0.95 (0.28; 1.62)	0.006
Mother’ s height	0.14 (0.10; 0.18)	<0.001
MPI score	−0.26 (−0.41; −0.12)	<0.001
**WAVZ**		
*Cryptosporidium spp.*	−0.43 (−0.80; −0.06)	0.023
Acute diarrhea	−0.56 (−0.82; −0.29)	<0.001
**LAVZ**		
*Cryptosporidium* spp.	−0.55 (−0.94; −0.17)	0.005
Mother’s height	0.02 (0.01; 0.03)	0.002

LAD: length-for-age difference, LAZ: length-for-age z-score, LAVZ: length velocity z-score, MPI: multidimensional poverty index, STH: soil transmitted helminths, WAVZ: weight velocity z-score, WLZ: weight-for-length z-score.

## Supplementary Materials

The following are available online at http://www.mdpi.com/1660-4601/15/4/688/s1, Table S1. Differences between infants who completed the study (at least 10 visits) and those who did not. Supplementary Table S2. Attained growth and growth velocity by age, during the study period. Supplementary Table S3. Univariable analysis for attained growth (WLZ, LAZ, and LAD). Supplementary Table S4. Univariable analysis for growth velocity (WAVZ and LAVZ).
