# Supplementary material for: Subclinical Enteric Parasitic Infections and Growth Faltering in Infants in São Tomé, Africa: A Birth Cohort Study
Source: Int J Environ Res Public Health. 2018 Apr 5;15(4):688. doi: 10.3390/ijerph15040688 (PMC5923730; doi:10.3390/ijerph15040688)
Supplement: Supplementary file 1 [file ijerph-15-00688-s001.pdf]

Table S1. Differences between infants who completed the study (at least 10 visits) and those who did not.

Variables	Complete follow-up	Incomplete follow-up	*p value* [a]
Sex, n (%)			
Female	142 (50.4)	100 (52.6)	0.627 [a]
Male	140 (49.6)	90 (47.4)	
District, n (%)			
Agua Grande	240 (85.1)	124 (65.3)	0.001 [a]
Caué	15 (5.3)	16 (8.4)	
Lembá	27 (9.6)	50 (26.3)	
Anthropometry at birth, mean (SD)			
Weight (Kg)	3.5 (0.48)	3.3 (0.46)	0.009 [b]
Length (cm)	50.3 (1.90)	49.9 (1.98)	0.050 [b]
Maternal data			
Mother's age, median (min-max)	26.0 (14–43)	24.0 (15–43)	0.009 [b]
Maternal height, mean (SD)	158.95 (5.9)	159.7 (5.8)	0.188 [c]
Mother's school years, median (min-max)	8.0 (0–15)	6.0 (0–15)	0.001 [b]
Improved sanitation			
Not, n (%)	95 (33.7)	98 (52.1)	0.001 [a]
Yes, n (%)	187 (66.3)	90 (47.9)	
Improved water source			
Not, n (%)	1 (0.4)	8 (4.3)	0.003 [a]
Yes, n (%)	281 (99.6)	180 (95.7)	

[a] *Chi-Square test,* [b] *Mann-Whitney Test,* [c] *t-test*

Table S2. Attained growth and growth velocity by age, during the study period.

Attained growth													Growth velocity *z*-scores					
Age	N	Weight (kg)		Length (cm)		WLZ		LAZ		LAD (Females)	LAD (Males)	Age	N	WAVZ		LAVZ	
		Mean	SD	Mean	SD	Mean	SD	Mean	SD	Mean	Mean			Mean	SD	Mean	SD
0–28 days	410	3.40	0.48	50.14	1.94	−0.01	0.99	−0.71	0.92	−1.52	−1.44	0–2 months	280	−0.22	0.98	−1.34	1.19
1 month	378	4.26	0.55	53.05	1.95	0.53	0.95	−0.72	0.93	−1.38	−1.26						
2 months	327	5.33	0.66	56.71	2.14	0.58	1.02	−0.65	0.95	−1.28	−1.25	2–4 months	275	0.06	1.08	0.18	1.23
3 months	336	6.13	0.71	59.93	2.05	0.38	0.96	−0.48	0.90	−0.74	−0.79						
4 months	320	6.74	0.81	62.37	2.18	0.32	1.03	−0.47	0.87	−0.98	−0.97	4–6 months	258	−0.32	1.11	−0.06	1.20
5 months	293	7.19	0.83	64.07	2.18	0.33	1.04	−0.55	0.91	−0.96	−0.93						
6 months	290	7.58	0.89	65.87	2.19	0.25	1.04	−0.49	0.90	−0.95	−0.89	6–8 months	211	−0.39	1.34	0.27	1.29
7 months	260	7.86	0.90	67.50	2.35	0.10	1.05	−0.44	0.92	−0.89	−0.60						
8 months	245	8.10	0.97	68.97	2.40	−0.03	1.03	−0.38	0.94	−0.87	−0.43	8–10 months	168	−0.66	1.30	−0.41	1.22
9 months	271	8.29	0.99	70.25	2.46	−0.15	1.04	−0.42	0.96	−0.81	−0.72						
10 months	234	8.54	0.98	71.59	2.45	−0.19	1.05	−0.41	0.94	−0.98	−0.76	10–12 months	214	−0.03	1.01	−0.33	1.20
11 months	254	8.62	1.01	72.52	2.55	−0.29	0.95	−0.50	0.97	−1.23	−0.78						
12 months	289	8.90	1.06	73.73	2.65	−0.27	0.99	−0.52	0.99	−1.47	−0.97	12–14 months	218	0.24	0.96	−0.21	1.01
14 months	243	9.47	1.08	75.88	2.66	−0.09	0.98	−0.56	0.96	−1.40	−0.86	14–16 months	220	0.28	1.07	0.00	0.97
16 months	282	9.89	1.03	78.01	2.74	−0.05	0.89	−0.55	0.96	−1.37	−1.36	16–18 months	238	−0.01	1.07	−0.12	0.96
18 months	265	10.30	1.07	79.80	2.87	0.00	0.85	−0.64	0.99	−1.70	−1.77	18–20 months	160	−0.46	1.23	0.03	0.98
20 months	180	10.62	1.10	81.84	3.06	−0.10	0.85	−0.60	10.00	−1.62	−1.68	20–22 months	112	−0.09	1.20	0.16	1.01
22 months	180	10.91	1.13	83.91	3.22	−0.30	0.81	−0.51	1.05	−0.62	−1.32	22–24 months	160	0.33	1.06	0.026	1.00
24 months	280	11.36	1.15	85.58	3.32	−0.20	0.82	−0.51	1.03	−1.52	−1.33						

LAD length –for-age difference; LAVZ length-for-age velocity z score; LAZ length-for-age z score; SD standard deviation; WAVZ weight-for-age velocity z score; WAZ weight-for-age z score; WLZ weight-for-length z score.

Table S3. Univariable analysis for attained growth (WLZ, LAZ, and LAD)

Variables	**WLZ**		**LAZ**		**LAD**	
	β-estimate (95% CI)	*p* value	β-estimate (95% CI)	*p* value	β-estimate (95% CI)	*p* value
Sex [a]	−0.01 (−0.16; 0.14)	0.892	0.05 (−0.11; 0.21)	0.521	0.08 (−0.28; 0.45)	0.661
MPI score	−0.05 (−0.10; −0.01)	0.028	−0.11 (−0.16; −0.06)	<0.001	−0.28 (−0.39; −0.16)	<0.001
Mother's height	−0.004 (−0.17; 0.01)	0.543	0.04 (0.03; 0.06)	<0.001	0.11 (0.08; 0.13)	<0.001
Exclusive breastfeeding	0.42 (0.19; 0.65)	<0.001	0.25 (0.01; 0.50)	0.040	0.59 (0.03; 1.15)	0.039
Duration breastfeeding	0.002 (−0.03; 0.03)	0.881	−0.03 (−0.06; 0.00)	0.052	−0.07 (−0.15; −0.002)	0.044
Age introduction complementary food	0.14 (0.05; 0.23)	0.003	0.01 (−0.09; 0.10)	0.864	0.03 (−0.19; 0.26)	0.766
Acute diarrhea	−0.31 (−0.38; −0.24)	<0.001	−0.06 (−0.12; −0.01)	0.023	−0.18 (−0.34; −0.01)	0.033
Acute respiratory infections	−0.14 (−0.18; −0.10)	<0.001	0.01 (−0.02; 0.04)	0.459	−0.05 (−0.14; 0.04)	0.260
Malaria	−0.34 (−0.60; −0.08)	0.010	0.04 (−0.15; 0.23)	0.700	−0.003 (−0.52; 0.51)	0.991
Infected	−0.19 (−0.26; −0.11)	<0.001	−0.09 (−0.15; −0.03)	0.003	−0.42 (−0.60; −0.24)	<0.001
Single infections	0.07 (−0.09; 0.24)	0.401	0.04 (−0.10; 0.18)	0.551	−0.02 (−0.48; 0.48)	0.948
Giardia lamblia	−0.18 (−0.28; −0.09)	<0.001	−0.11 (−0.19; −0.04)	0.004	−0.46 (−0.70; −0.23)	<0.001
Cryptosporidium spp.	−0.12 (−0.28; 0.03)	0.124	−0.01 (−0.14; 0.11)	0.814	−0.10 (−0.48; 0.28)	0.617
STH	−0.13 (−0.23; 0.02)	0.017	−0.16 (−0.24; −0.07)	<0.001	−0.60 (−0.85; −0.34)	<0.001

[a] Reference category: female, CI confidence interval, LAD length-for-age difference, LAZ length-for-age z-score, MPI multidimensional poverty index, STH soil transmitted helminths, WLZ weight-for-length z-score.

Table S4. Univariable analysis for growth velocity (WAVZ and LAVZ)

Variables	WAVZ		LAVZ	
	β-estimate (95% CI)	*p* value	β-estimate (95% CI)	*p* value
Sex [a]	−0.06 (−0.15; 0.04)	0.254	0.002 (−0.10; 0.10)	0.966
MPI score	−0.03 (−0.06; −0.004)	0.027	−0.03 (−0.06; −0.004)	0.025
Mother's height	0.01 (0.003; 0.02)	0.009	0.01 (0.01; 0.02)	0.001
Exclusive breastfeeding	−0.02 (−0.15; 0.10)	0.719	−0.08 (−0.21; 0.05)	0.231
Duration breastfeeding	−0.01 (−0.03; 0.01)	0.183	−0.01 (−0.02; 0.01)	0.416
Age introduction complementary food	0.002 (−0.05; 0.05)	0.915	0.02 (−0.04; 0.07)	0.516
Acute diarrhea	−0.43 (−0.60; −0.25)	<0.001	0.05 (−0.13; 0.24)	0.587
Acute respiratory infections	−0.05 (−0.15; 0.06)	0.364	0.06 (−0.05; 0.17)	0.291
Malaria	−0.04 (−0.69; 0.62)	0.911	0.94 (0.26; 1.62)	0.007
Infected	0.05 (−0.12; 0.22)	0.547	−0.08 (−0.25; 0.09)	0.378
Single infections	−0.21 (−0.58; 0.17)	0.275	0.19 (−0.15; 0.54)	0.271
Giardia lamblia	0.11 (−0.10; 0.32)	0.307	−0.06 (−0.27; 0.16)	0.609
Cryptosporidium spp.	−0.43 (−0.81; −0.06)	0.025	−0.66 (−1.05; −0.28)	0.001
STH	0.18 (−0.04; 0.41)	0.115	0.01 (−0.22; 0.25)	0.912

[a] Reference category: female, CI confidence interval, LAVZ length velocity z-score, MPI multidimensional poverty index, STH soil transmitted helminths, WAVZ weight velocity z-score.
